# Hosts and vectors of scrub typhus in Chile: epidemiological study and molecular analyses of *Orientia* infection in rodents and rodent-associated mites

**DOI:** 10.1186/s13071-024-06602-0

**Published:** 2024-12-18

**Authors:** Constanza Martínez-Valdebenito, Gerardo Acosta-Jamett, Rayitray Abello, Ju Jiang, Allen L. Richards, Katia Abarca, Thomas Weitzel

**Affiliations:** 1 Departamento de Enfermedades Infecciosas e Inmunología Pediátricas, Escuela de Medicina, Pontificia Universidad Católica de Chile, Santiago, Chile; 2 Instituto de Medicina Preventiva Veterinaria and Center for Disease Surveillance and Evolution of Infectious Diseases, Facultad de Ciencias Veterinarias, Universidad Austral de Chile, Valdivia, Chile; 3 Naval Medical Research Center, Silver Spring, MD USA; 4 Uniformed Services University of the Health Sciences, Bethesda, MD USA; 5 Laboratorio Clínico, Facultad de Medicina Clínica Alemana, Clínica Alemana de Santiago, Universidad del Desarrollo, Santiago, Chile; 6 Instituto de Ciencias e Innovación en Medicina (ICIM), Facultad de Medicina Clínica Alemana, Universidad del Desarrollo, Santiago, Chile

**Keywords:** Scrub typhus, Vector-borne diseases, Zoonotic diseases, Rickettsiales, Epidemiology, South America

## Abstract

*Candidatus* Orientia chiloensis causes scrub typhus over a wide geographical range in southern Chile. The life cycle, including vectors and reservoirs of this novel rickettsial pathogen, is incompletely understood. We analyzed rodent tissue and rodent-associated mite samples collected during a field study in six localities on Chiloé Island, where human scrub typhus cases have occurred. Using molecular methods, we detected *Orientia* DNA in 24.8% of rodents, belonging to five of seven captured species. *Orientia*-infection rates showed geographical variations, but were not influenced by rodent species, sex, age, and mite infestation. Phylogenetic analysis showed that *Orientia* sequences from trombiculid mites (*Proschoengastia eloisae*) were identical to those from scrub typhus patients from the same region. The results suggest that these rodent-associated mites serve as vectors and play an important role in the ecology of scrub typhus in southern Chile. Further studies are required to determine whether *Orientia*-infected rodents can also serve as reservoir of *Orientia* in Chile.

**Graphical Abstract:**

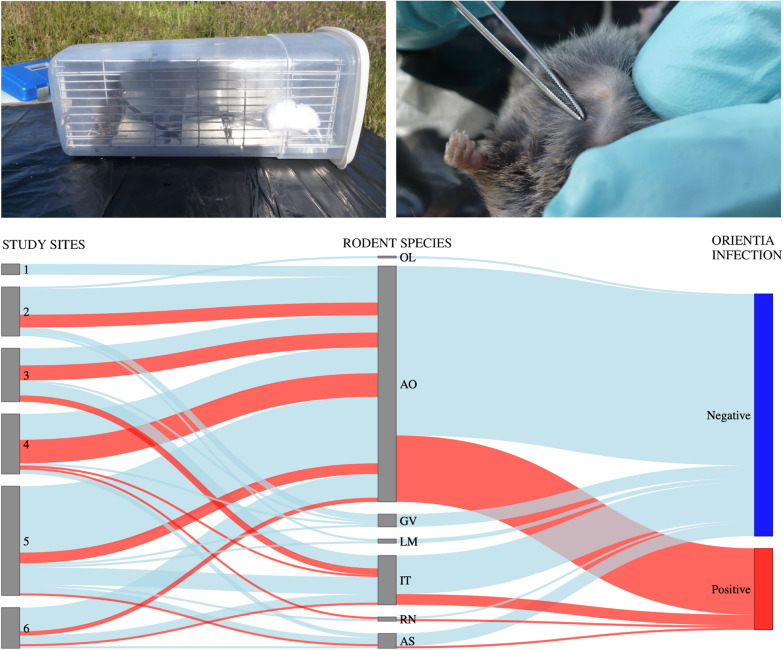

**Supplementary Information:**

The online version contains supplementary material available at 10.1186/s13071-024-06602-0.

## Background

Scrub typhus is a vector-borne disease caused by intracellular bacteria of the genus *Orientia*, Rickettsiaceae family, which manifests as an acute and potentially severe febrile illness [1]. Thus far three *Orientia* species have been described as human pathogens: (1) *Orientia tsutsugamushi* in Asia–Pacific (> 1 million annual cases) [1]; (2) *Candidatus* Orientia chuto on the Arabian Peninsula (one patient) and in Uganda (one patient) [2, 3], and (3) *Candidatus* Orientia chiloensis, with > 150 confirmed cases and a recent outbreak in southern Chile [4]. Furthermore, serological surveys as well as rodent and vector studies have suggested that *Orientia* spp. might have a much wider geographical distribution, including Africa, Europe, and North America [5, 6].

In the Asia–Pacific region, trombiculid mites of the genus *Leptotrombidium* serve as vector and reservoir of *O. tsutsugamushi* [6]. In other regions, however, the life cycle of orientiae is uncertain. Recently, *Ca.* O. chuto was detected in trombiculid mites collected from rodents in Kenya and Saudi Arabia [7, 8]. Rodent studies in regions with endemic scrub typhus in Chile identified known and previously unknown trombiculid mite species [9–11]. In the Los Lagos Region, several species tested positive for *Orientia* DNA by *Orientia*-specific quantitative polymerase chain reaction (qPCR, Orien16S), suggesting their possible role as vector and reservoir [9, 11]. In the Aysén Region, a distinct species, *Proschoengastia antarctica* (formerly in *Herpetacarus*) [12], was confirmed as vector of scrub typhus in 2022 [13]. The presence of *Orientia* spp. in animals in Chile has not been studied yet, except for a serological survey of dogs, a known susceptible species of scrub typhus [14]. The present work firstly investigated the prevalence of *Orientia* infection in rodents in southern Chile, analyzed associated factors, and compared *Orientia* sequences from mites collected from the rodents to those of patients with Chilean scrub typhus.

## Methods

During the summer months of January–March 2018, rodents were trapped and examined for mite infestation in six sites on Chiloé Island, as previously reported [8]. During the field project, captured rodents were euthanized by cervical dislocation after anesthesia with isoflurane, and tissue samples of approximately 1 cm^3^ (lung, spleen, and kidney) were obtained by dissection, stored in liquid nitrogen, and transported on dry ice to Santiago. There, tissue specimens from the same animal were pooled and homogenized using silica beads in 1 mL PBS 1X in five cycles of 1 min in a homogenizer (BeadBug 6, Benchmark Scientific D1030-E). Then, DNA was extracted from 400 µL of the homogenized tissue using an automated extractor, MagNa Pure 24 (Roche Diagnostics, IN, USA), eluted in 50 µL of buffer (MagNA Pure External Lysis Buffer; Roche Diagnostics), and analyzed for the presence of *Orientia* spp. DNA by Orien16S, an *Orientia*-specific real-time PCR protocol targeting the 16S rRNA gene (*rrs*) [15].

Rodent-associated trombiculid mites were collected and identified as described in detail in Acosta-Jamett et al. [9]. In brief, mites were collected from each rodent’s body surface by brushing over a plate with water; furthermore, the skin of euthanized animals was stored in 95% ethanol and later revised using a magnifying glass for additional chiggers. Mite samples of individual rodents were separated by morphotype and stored in 95% ethanol. One specimen of each morphotype, collected from each individual rodent, was cleared in Nesbitt’s solution, mounted in Berlese’s medium, and microscopically identified. Then, DNA from pools of 6–20 mites of the same mite species, deriving from an individual rodent, was extracted after disruption by a freeze–thaw cycle, as previously described [9]. Total DNA was then automatically extracted using the MagNA Pure System to a final elution volume of 50 μL. Each PCR run included a negative control (PCR water) and a positive control (plasmid control, as described in Jiang et al. [15]), which were loaded at the same time as each sample tested.

For statistical analyses we calculated the prevalence rates of *Orientia*-infected rodents and performed a univariate analysis for associated factor using logistic regression for the following parameters: trapping site (no. 1–6), rodent species, rodent sex (male, female), rodent age (juvenile, adult), and infestation with trombiculid mites. All analyses were carried out in R (version 4.1.0, https://www.r-project.org/).

To obtain larger *Orientia* sequences from *Orientia*-positive rodent tissue and from mite pools, which tested *Orientia*-positive by qPCR and identified as *Proschoengastia eloisae* in previous studies [9, 10], we applied a modified hemi-nested PCR targeting *rrs*, as previously applied for human samples [16]. Details of the primers can be found in Supplementary Table S1. In short, we used the primer pair 16SO79 and 16sOR1198R for the first PCR reaction and 16s155F and 16sOR1198 for the second PCR step. To improve sensitivity, we added a third reaction using primers of the second PCR step. For each PCR we utilized the Platinum PCR SuperMix High Fidelity (cat. 12532016) for the second and third PCR utilizing 2.5 µL of the previous PCR product. The final amplified segment was sequenced by Macrogen USA (Maryland, USA) using the primers 16s155F and 16sOR1198. To avoid false positives, a negative control was included at the start of the first run and held until the final PCR round. In addition, new negative controls were added at the beginning of each PCR and one at the end, to control for possible spillover during the loading process. As extra measures, gloves were changed after opening of tubes for loading, the PCR workstation was cleaned and exposed for 15 min to UV light after each load, and separate rooms were used for extraction, sample loading, and PCR reactions.

For the phylogenetic analysis we compared the sequences from this study and sequences of 17 *Ca.* *O. chiloensis* samples from scrub typhus patients of a previous study [16] and compared them with sequences from GeneBank of *O. tsutsugamushi* (*n* = 15), *Ca.* *O. chuto* (*n* = 1), and *Rickettsia* spp. (*n* = 3), as well as other bacterial species (*n* = 2). Sequences were aligned by BioEdit version 7.0.5.3 (http://www.mbio.ncsu.edu/bioedit/bioedit.html) using ClustalW (http://www.clustal.org). The phylogenetic analysis was inferred by maximum likelihood (ML) method using the software MEGAX [17]. The search of the most appropriate model of nucleotide substitution for phylogenetic analysis was performed according to the Bayesian information criterion (BIC). Initial trees for the heuristic search were obtained automatically, applying NJ and Bio NJ algorithms to a matrix of pairwise distances estimated using the maximum composite likelihood approach, and then selecting the topology with superior log likelihood value.

## Results

**Fig. 1:**
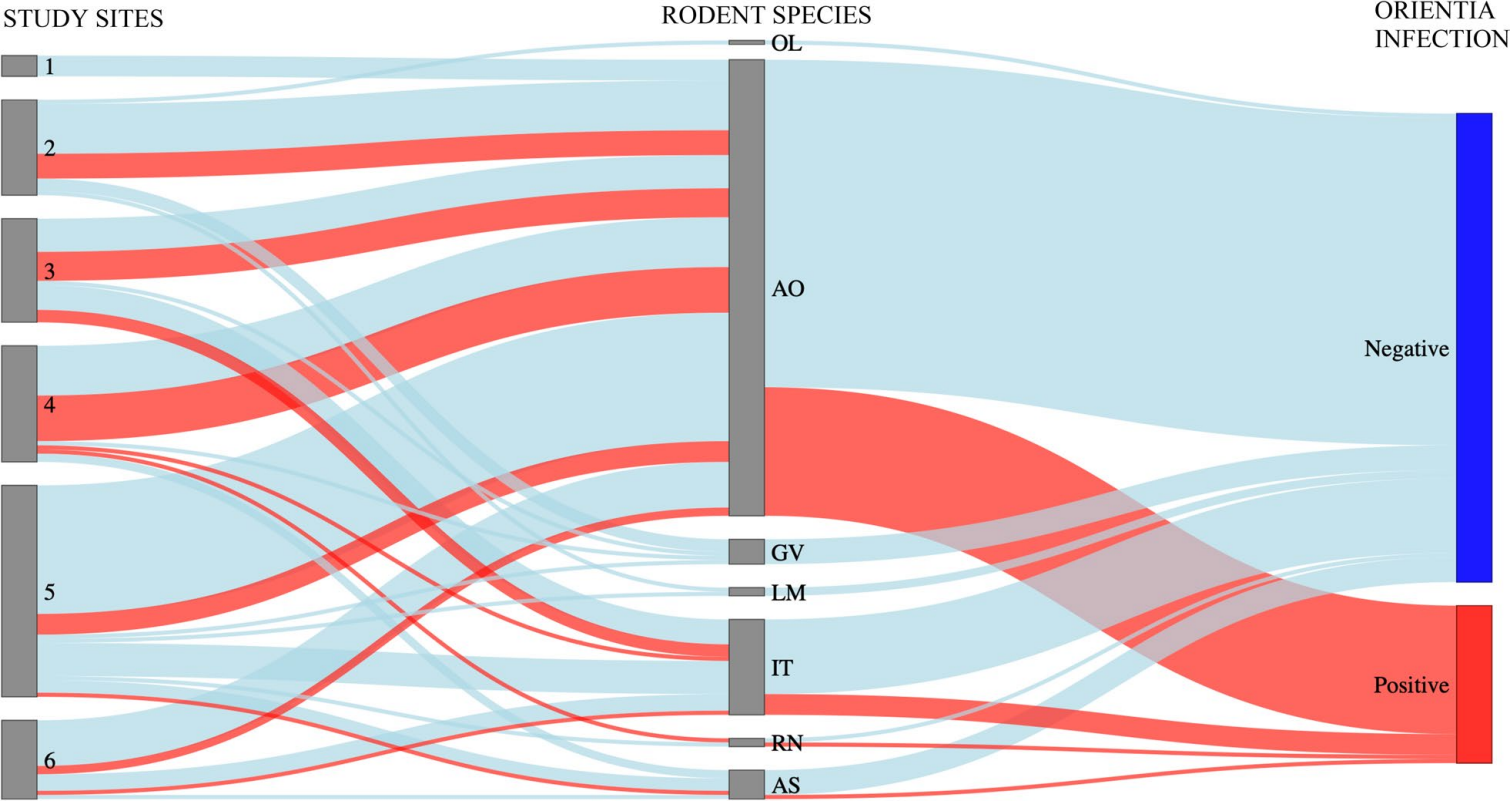
Sankey diagram showing distribution of *Orientia* infection (red, positive; blue, negative) among different rodent species captured in six study sites (1–6) on Chiloé Island (*n* = 151). OL, *Oligoryzomys longicaudatus*; AO, *Abrothrix olivacea*; GV, *Geoxus valdivianus*; LM, *Loxodontomys micropus*; IT, *Irenomys tarsalis*; RN, *Rattus norvegicus*; AS, *Abrothrix sanborni*

Rodents infected with *Orientia* spp. were detected in five of six study sites, belonging to four of the seven identified species (Table 1). Of the 153 examined rodents, 38 were positive for *Orientia* DNA, resulting in an overall prevalence of 24.8% (95% CI 18.7–32.3%). As visualized in Fig. 1, *Orientia* infection was widely distributed and did not cluster in certain rodent species or at certain sites. Among analyzed factors for *Orientia* infection in rodents, rodent species, sex, age group, and chigger infestation did not significantly affect *Orientia* positivity (Table 1). Univariate analysis of sampling sites indicated a significant difference (*P* < 0.05) between sites 4 and 6; however, the wide and overlapping 95% confidence intervals indicated that this finding might have been affected by low numbers of infected rodents. Of the 38 rodents that tested *Orientia*-positive, 23 (60.5%) were mite infested and 6/23 (26.1%) had *Orientia*-positive mites (see Fig. S1). Among *Orientia*-negative rodents, 70/115 (60.9%) were infested by mites and 12/70 (17.1%) had *Orientia*-positive mites (Fig. S1). Overall, among the 50 rodents with *Orientia* DNA in tissue samples or infesting mites, only six (12.0%) showed concordant results, i.e., *Orientia*-infection in both tissue samples and infesting mites (Fig. S1).

**Table 1:** Univariate logistic regression analysis of factors associated with *Orientia* infection among 153 rodents captured on Chiloé Island

Risk factor	*N*	*Orientia* infection				
		*n*	%	95% CI	Odds ratio	*P*-value
Study site						
No. 6	19	3	15.8	5.7–37.9	1.00	
No. 5	53	6	11.3	5.4–22.6	0.68	0.615
No. 4	28	13	46.4	29.5–64.3	4.62	0.037*
No. 3	25	10	40.0	23.4–59.4	3.56	0.091
No. 2	23	6	26.1	12.6–47.1	1.88	0.422
No. 1	5	0	0.0	0.0–45.9	NA	0.989
Rodent species						
* Abrothrix olivacea*	110	31	28.2	20.6–37.2	1.00	
* Irenomys tarsalis*	23	5	21.7	9.8–42.2	0.71	0.529
* Geoxus valdivianus*	6	0	0.0	0.0–41.0	NA	0.992
* Rattus norvegicus*	2	1	50.0	9.4–90.6	2.55	0.513
* Abrothrix sanborni*	7	1	14.3	3.2–52.7	0.42	0.437
* Loxodontomys micropus*	2	0	0.0	0.0–70.8	NA	0.995
* Oligoryzomys longicaudatus*	1	0	0.0	0.0–84.2	NA	0.997
Missing data	2	0	0.0			
Rodent sex						
Female	21	5	23.8	10.7–45.4	1.00	
Male	132	33	25.0	18.4–33.0	1.07	0.882
Rodent age						
Juvenile	89	22	24.7	16.9–34.6	1.00	
Adult	64	16	25.0	16.0–36.9	1.02	0.968
Mite infestation^a^						
Without	60	15	25.0	15.8–37.3	1.00	
Any trombiculid	93	23	24.7	17.1–34.4	0.99	0.970

95% CI, 95% confidence interval; NA, not applicable

^a^Further details of trombiculid species and infection rates can be found in Tables 1 and 2, Acosta-Jamett G et al. [9]

^*^Statistically significant

**Fig. 2:**
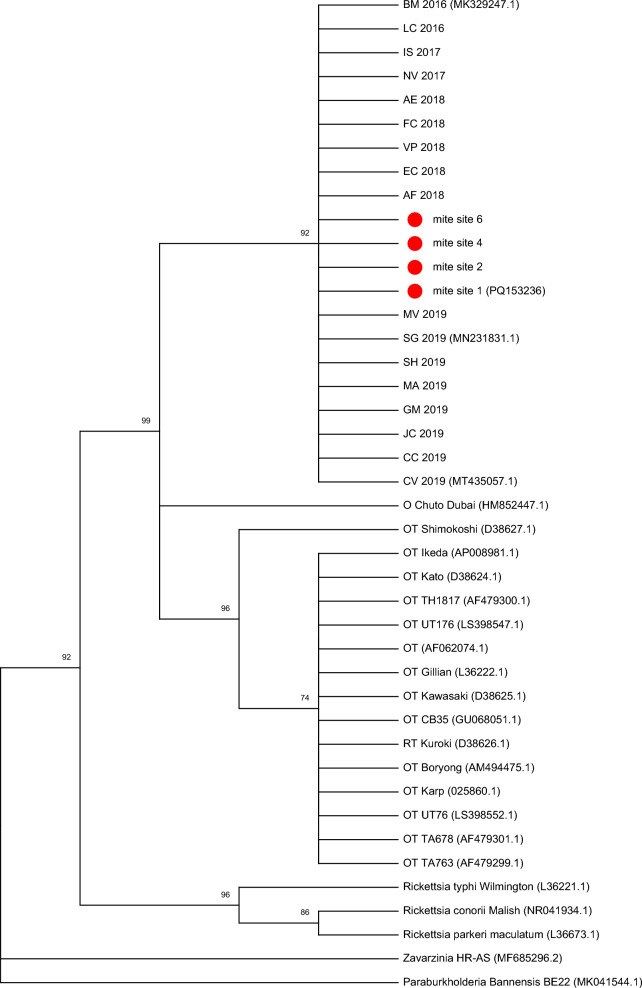
Phylogenetic tree of 16S RNA gene (*rrs*) of *Candidatus* Orientia chiloensis (OC) from trombiculid mites and patients with scrub typhus, as well as *Orientia tsutsugamushi* strains (OT), *Candidatus* Orientia chuto, and other bacteria. The evolutionary history was inferred by using the maximum likelihood method and Tamura-Nei model [18]. The tree with the highest log likelihood (−2747.08) is shown. The percentage of trees in which the associated taxa clustered together is shown next to the branches. Initial tree(s) for the heuristic search were obtained automatically by applying Neighbor-Join and BioNJ algorithms to a matrix of pairwise distances estimated using the maximum composite likelihood (MCL) approach, and then selecting the topology with superior log likelihood value. A discrete Gamma distribution was used to model evolutionary rate differences among sites [five categories (+ *G*, parameter = 0.1974)]. This analysis involved 42 nucleotide sequences. All positions with less than 95% site coverage were eliminated, i.e., fewer than 5% alignment gaps, missing data, and ambiguous bases were allowed at any position (partial deletion option). There was a total of 859 positions in the final dataset. Evolutionary analyses were conducted in MEGA X [19]. Red circles indicate the sequences from trombiculid mites of this study. Sequences retrieved from GenBank have the genus, followed by the species, strain, and GenBank accession number. Sequences from the different *Orientia tsutsugamushi* strains are named OT, followed by the strain

The sequencing protocol was applied to all qPCR-positive rodent samples and to the *Orientia*-positive mite pools, which had the lowest Ct value per study site [9]. The four selected pools derived from sites 2, 4, 5, and 6, where they had been collected from *Geoxus valdivianus*, *Abrothrix olivacea*, *Abrothrix sanborni*, and *Abrothrix olivacea*, respectively. As previously reported [9], all positive mite pools belonged to the species *P. eloisae* (described in *Herpetacarus*) [12]. Ct values of the four mite samples were 33.38, 30.94, 33.72, and 30.57, respectively. Only *Abrothrix olivacea* from site 4 was also tested positive in tissue. All four selected pools were successfully amplified, sequenced, and analyzed. The generated sequences (875 bp) were 100% identical to each other and to previously published sequences of 17 patients with Chilean scrub typhus [16] (Fig. 2, Supplementary Table S2). The sequence from mite pool no. 1 was submitted to GenBank (accession number PQ153236). Intents to generate amplicons for sequencing from tissue samples, which were positive by qPCR, were unsuccessful, although real-time PCR was repeated by another technician. In addition, qPCR results were verified by sequential testing, excluding stochastic amplification, which might occur after > 30 amplification cycles [17]. All sequential runs of samples with Cts > 35 cycles were positive after 20–25 cycles, demonstrating that signals represented true amplicons.

## Discussion

The paradigm of scrub typhus as a disease caused by a single *Orientia* species (*O. tsutsugamushi*), transmitted by *Leptotrombidium* mites, has shifted in recent years [5]. However, eco-epidemiological studies on newly discovered *Orientia* species and their vectors are complex and yet scarce. Until now, our research group has detected > 150 patients with scrub typhus over a distance of more than 2000 km in southern Chile (Chilean Rickettsia and Zoonosis Research Group, unpublished data). Molecular analyses demonstrated that the infection in Chile is caused by *Ca.* O. chiloensis [16]. Rodent studies from Chiloé Island showed that 17% of pooled samples of *P. eloisae*, a novel mite species collected from rodents, contained *Orientia* DNA [9]. The present additional analyses of rodent and mite samples from this field study provide further insight into the ecology of this emerging pathogen.

The present study showed that about 25% of the captured rodents carried *Orientia* DNA fragments. As shown in Fig. S1, only 6 of 38 (15.8%) *Orientia*-positive rodents were parasitized by *Orientia*-infected mites at time of capture. The presence of such DNA fragments in the absence of infected mites is compatible with prolonged or persistent *Orientia* infection, as observed in Asia–Pacific, where *O. tsutsugamushi* infected rodents for up to 4 months [20]. Figure 1 demonstrates that the presence of *Orientia* DNA was widespread, indicating that various rodent species at different sites carried the bacterium. However, the capacity of rodents and other vertebrates to serve as reservoir or amplifying host of *Orientia* spp. is uncertain. Different to most other arthropod vectors, trombiculid mites feed only once within their life cycle. As experimental studies have shown, the ingestion of *O. tsutsugamushi* from infected vertebrates does not lead to a sustained vertical propagation of *Orientia* within chigger populations. This implicates that the mites are the exclusive reservoir for the infection. However, as stated by Elliott and colleges in their comprehensive review, these findings were mostly obtained in artificial mite colonies and with low numbers of mites, and therefore do not necessarily represent the situation in nature [20]. Since in Chile both mite species and *Orientia* species differ from Asia–Pacific, the capacity and role of rodents to serve as reservoir are uncertain and require further studies.

The detection of *Orientia*-positive mites collected from *Orientia*-negative rodents is also of notice. Since trombiculid mites only parasitize once during their lifecycle, this finding implicates that the larvae were vertically infected, and strengthens the assumed role of *P. eloisae* as vector and reservoir of *Ca.* O. chiloensis. This hypothesis is supported by additional data from site no. 3, where in the present study *Orientia* was only present in rodents, but not in the identified mite species *Paratrombicula goffi* and *Quadraseta chiloensis*. In a more recent field project of our group at the same locality (site no. 3), however, *Orientia* was detected only in mites of the species *P. eloisae* [11], indicating a likely role of this species in the ecology of *Ca.* O. chiloensis in this region. In addition, our study provides molecular evidence for the transmission of *Ca*. O. chiloensis by *P. eloisae*, since *Orientia* sequences obtained from this mite species were identical to those from clinical samples of Chilean cases of scrub typhus. The vector and reservoir capacity of another species within the same genus, *P. antarctica*, was demonstrated about 650 km south of Chiloé Island in the Aysén Region [13]. Further studies should focus on determining whether the genus *Proschoengastia* plays a similar role in Chile for transmitting and maintaining *Orientia*, as the *Leptotrombidium* genus does in the Asia–Pacific region.

A limitation of the study is that sequencing of longer *Orientia* amplicons from rodent tissue was not successful. The amplification of longer sequences from tissue samples is challenging due to their high nuclease activity, leading to rapid degradation, as recently shown in an experimental study [21]. Poor performance of rodent tissue was also reported in a sequencing study from Thailand [22]. In our project, rodents were dissected under BSL-3 field conditions during summer, placed into liquid nitrogen in the field, and then transported on dry ice to Santiago (distance > 1000 km) [9]. The complexity of this dissection-to-laboratory phase might also have contributed to DNA degradation. The high variability of *Orientia* prevalence in rodent studies from Asia–Pacific might partly be caused by such technical and methodological challenges [20, 22–25].

In summary, our study showed that tissue samples from various rodent species captured on Chiloé Island carried *Orientia* DNA; the phylogenetic analysis of *Orientia* DNA from rodent-associated mites suggests their role as possible vectors and reservoir of this pathogen in this region.

## Supplementary Information

Additional file 1: Table S1. Primers and probes. Sequences and references of the primers used for molecular analyses. All primers are designed against 16sRNA gene (*rrs*); numbers after hyphen indicate the primer position in the gene.

Additional file 2: Table S2. Identity Matrix. The matrix displays the similarities of 42 sequences of the 16S RNA gene (*rrs*), obtained from *Candidatus* Orientia chiloensis (from trombiculid mites and scrub typhus patients), as well as *Orientia tsutsugamushi* strains, *Candidatus* Orientia chuto, and other bacteria. Sequences were align using ClustalW tool within Bioedit, later the matrix was retrieved from Bioedit tool.

Additional file 3: Figure S1. Venn diagram of rodents captured on Chiloé Island (*n* = 153) grouped by mite infestation and presence of *Orientia* DNA in rodent tissue and in mites.

## Data Availability

The data supporting the findings of this study are available within the paper and its Supplementary Information. Further details on methodology and results are available from the corresponding authors upon reasonable request.

## Declarations

### Disclaimers

The views expressed in this article are those of the authors and do not necessarily reflect the official policy or position of the Department of the Navy, Department of Defense, nor the U.S. Government. At the time of the investigation, A.L.R. was an employee of the U.S. Government, and this work was prepared as a part of his official duties. Title 17 U.S.C. §105 provides that ‘Copyright protection under this title is not available for any work of the United States Government.’ Title 17 U.S.C. §101 defines a U.S. Government work as a work prepared by a military service member or employee of the U.S. Government as part of that person’s official duties.
