# Supplementary material for: Hosts and vectors of scrub typhus in Chile: epidemiological study and molecular analyses of Orientia infection in rodents and rodent-associated mites
Source: Parasit Vectors. 2024 Dec 18;17:514. doi: 10.1186/s13071-024-06602-0 (PMC11658049; doi:10.1186/s13071-024-06602-0)
Supplement: Supplementary file 1 — Additional file 1: Table S1. Primers and probes. Sequences and references of the primers used for molecular analyses. All primers are designed against 16sRNA gene (rrs); numbers after hyphen indicate the primer position in the gene. [file 13071_2024_6602_MOESM1_ESM.pdf]

S2 Table. Primers and probes.

Primer/probe name	Sequence	Reference
16s-563F	GCCTGATCCAGCAATG	15
16S-656R	GGCTTTTTCTGTAGGTAC	
16S-636	TCATTATCATCCCTACTAAAAGAGCTTTACA	
16S079F	ATTAATGCTGAGCTTGCTTAGCAT	16
16SOR1198R	TTTCCTATAGTTCCCGGCATT	
16SOR155F*	TCAGTACGGAATAACWTTTAGAAATAA	

*used with 16SOR1198R
