# Supplementary figures and images for: Hosts and vectors of scrub typhus in Chile: epidemiological study and molecular analyses of Orientia infection in rodents and rodent-associated mites
Source: Parasit Vectors. 2024 Dec 18;17:514. doi: 10.1186/s13071-024-06602-0 (PMC11658049; doi:10.1186/s13071-024-06602-0)

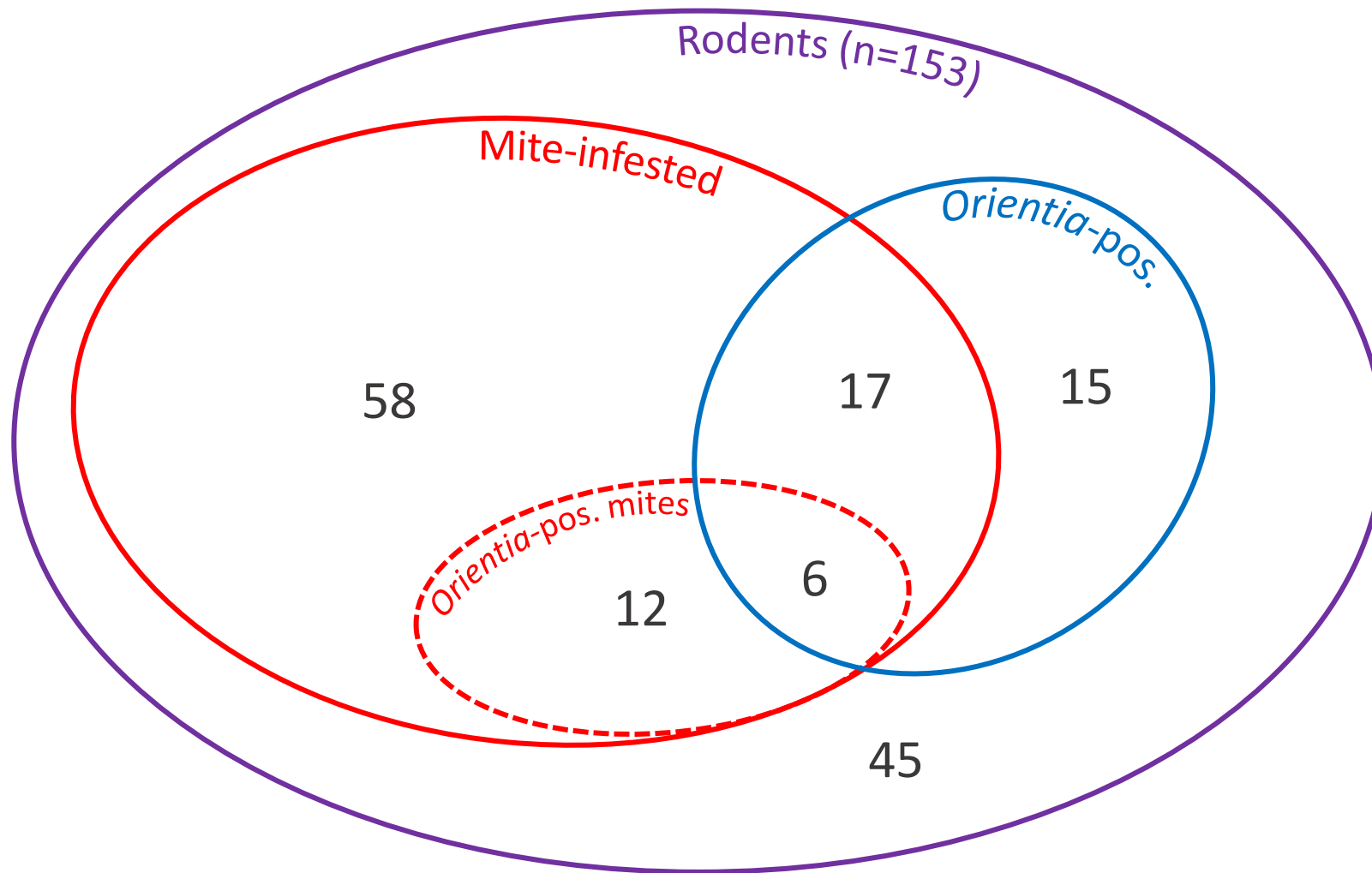

Supplement: Supplementary file 3 — Additional file 3: Figure S1. Venn diagram of rodents captured on Chiloé Island (n = 153) grouped by mite infestation and presence of Orientia DNA in rodent tissue and in mites. [file 13071_2024_6602_MOESM3_ESM.pdf]
